# The Hospital World

**Published:** 1910-05-21

**Authors:** 


### THE HOSPITAL WORLD.

### Elections and Appointments.

Lady Bruce has been elected President of the Ladies' Committee of the Nottingham Children's Hospital.

Mr. Roe has been elected as the working men's representative on the monthly board of the Malton Cottage Hospital.

The Duke of Wellington again has been elected Chairman of the Royal Hants County Hospital; Major Alexander is again Treasurer

Mr. Whitbread has been re-elected President of the Bedford County Hospital, and Mr. T. H. Barnard has been reappointed Treasurer.

The Committee of Management of the Royal Hants County Hospital consists of Mr. J. C. Warner, the Rev. Canon Martin, the Rev. J. M. Freshfield, Mr. R. J. Harris, Captain Hughes, R.N., Mr. L. B. Keyser, Colonel Whitmore Smith, Colonel Cockburn, D.S.O., Mr. W. O. F. Sergeant, Admiral Dicken, Mr. W. Douglas Bishop, and the Rev. C. H. H. Cooper. Sir William Portal, Judge Gye, Mr. S. Bostock, and Mr. H. Nicoll have been re-elected Governors on the Committee of Selection.

Sir Lees Knowles, Bart., has resigned the presidency of the Royal Salford Hospital, and a special meeting of the Board of Management has been held to elect his successor. Sir William Mather was unanimously elected, Mr. E. G. Wood and Sir William Stephens declaring that he was pre-eminently the best candidate, Mr. Wood reminding the meeting that Sir William Mather was "connected with every society in the district which had for its object the uplifting of the poor."

Mr. John Kendall has been elected Chairman of the newly constituted Sir Titus Salt's Hospital, Saltaire, Bedford. In taking the chair, Mr. Kendall remarked: "I do not in the least desire to monopolise the position, but so long as you wish me to take this position I shall consider it an honour to do so. Before the hospital was enlarged there was much criticism of our intentions, but if the critics could see the building as it is now I do not think there would have been much criticism."

The General Committee of the Benn Ulster Eye, Ear, and Throat Hospital, Belfast, has been re-elected as follows: Mr. C. W. Dunbar-Buller, D.L.; Lieut.-Col. M'Farland, A.M.D.; Rev. F. J. M'Neice, B.D.; Rev. Saml. Thompson, M.A.; Mr. Henry Hutton, J.P.; Mr Henry Seaver, C.E.; Mr. Henry Macaulay, Mr. Wm. R. Rea, Mr. Frank Workman, Mr. James S. Reid, and Mr. W. H. Orr.

The Committee of Management of the Nottingham Eye Hospital has been elected as follows: Alderman J. P. Ford, Dr. W. G. Laws, Dr. H. Herbert, Dr. T. Henderson, Mr. A. Heymann, Mr. A. Armitage, Mr. G. H. Stubington, Mr. W. Bradshaw, Mr. J. N. Derbyshire, Mr. S. Armitage, Mr. A. Eberlin, Mr. H. J. Weinberg, and Mr. R. Halford.

### Personalia.

It is no small compliment to the energetic ability of Mr. F. B. Ray that the Tolworth Isolation Joint Hospital Board should so far depart from precedent as to re-elect him its chairman. The administrative improvements in the hospital which have marked the past year or two have been due in no small degree to Mr. Ray's powers as a worker.

When a hospital leaves the strictly business track its activities necessitate, what the world calls a man of business is the best friend it can have. The Royal Mineral Water Hospital, Bath, then, has a genuine loss in the resignation of Mr. J. E. G. Bradford from the Estates Committee. This Estates Committee runs, we believe, the hospital's landed property at Charmey Down, and, in the Rev. C. E. Barnwell's words, "his knowledge of law has been exceedingly useful to the committee." It is good to learn that Colonel Kirkwood is to succeed him, for the Colonel is a most useful go-between for County Council and hospital; at this time, too, when the Council is about to become the hospital's lessee for some twenty-two acres from the Charmey Down estate a common representative will be invaluable.

Nothing is more inspiring to the student of institution history than the way in which families often cluster round institutions, and from generation to generation bear their part in the hospital's work. In this way the institution becomes the centre of traditions, the parent tree round which the genius of one or more families expends its force. This is how the hospital spirit is born and how it grows in time into a great tradition, lurking, as it were, in the very crevices of the hospital's walls. An example of this may be found in the records of the Royal Mineral Water Hospital, Bath. That hospital had a rather heavy death-roll last year among its governors, and the long connection of the name of one of them particularly with the institution is an inspiring record for posterity. When Major-General Ralph Allen died, five generations were completed during which the family he represented had grown intertwined with the hospital's life. The hospital was established in 1737, and incorporated in 1739 by Act of Parliament. In the 173 years of its existence, only three intervals have occurred during which the name of Allen has been absent from the roll of governors. What a record for the Royal Mineral Water Hospital! What a fine thing for the Allen family too, for we venture to suggest that it is not only the institution which gains from such connections as these. Perhaps there is a subtler influence than the mineral waters at Bath to account for these things. A glance at the list of late governors shows that Mr. T. F. Inman held office for 37 years, that Colonel Brymer had been a governor for 27, and there are others with almost as long a record. The only thing which can account for this is the hospital spirit. Of the way in which it may be created elsewhere the above record is full of suggestion.

